# Modulation of* mec*A Gene Expression by Essential Oil from* Salvia sclarea* and Synergism with Oxacillin in Methicillin Resistant* Staphylococcus epidermidis* Carrying Different Types of Staphylococcal Chromosomal Cassette* mec*

**DOI:** 10.1155/2016/6475837

**Published:** 2016-01-06

**Authors:** Romana Chovanová, Mária Mikulášová, Štefánia Vaverková

**Affiliations:** ^1^Department of Molecular Biology, Faculty of Natural Sciences, Comenius University, Mlynská Dolina, Ilkovičova 6, 842 15 Bratislava, Slovakia; ^2^Department of Pharmacognosy, Faculty of Pharmacy, Comenius University, Odbojárov 10, 832 32 Bratislava, Slovakia

## Abstract

The essential oil (EO) from* Salvia sclarea* was shown to increase the susceptibility of methicillin resistant* Staphylococcus epidermidis* (MRSE) isolates to oxacillin. The purpose of this study was to investigate the effect of EO from* S. sclarea* on expression of* mec*A gene of MRSE carrying different types of staphylococcal chromosomal cassette (SCC*mec*) and to evaluate potential synergistic effect of EO with oxacillin. Using real-time PCR we found that EO alone inhibited the expression of the resistant genes* mec*A,* mec*R1, and* mec*I and* bla*Z,* bla*R1, and* bla*I. The use of the combination of EO with oxacillin resulted in significantly inhibited expression of* mec*A gene in all tested strains with different types of SCC*mec*. Using time-kill assay and checkerboard assay we confirmed synergistic effect of EO from* S. sclarea* and oxacillin in MRSE.

## 1. Introduction

The antibiotic resistance provides a great therapeutical and economic burden in the treatment of infectious diseases and it may threaten the success of antimicrobial chemotherapy. The disproportion between the slow development of new drugs and the fast emergence of resistant strains necessitates the development and research of new antimicrobial agents or resistance modifiers. One strategy employed to overcome resistance mechanisms is a combination therapy such as using clavulanic acid as inhibitor of *β*-lactamase in drugs sulbactam and tazobactam [1]. However, the frequent use of clavulanate has led to the emergence of resistant bacterial strains [2]. The promising strategy is the use of synergistic effects of natural compounds, products of plant secondary metabolism. Traditionally, medicinal plants have been used throughout the world for centuries for a range of medicinal complications. Plant drugs are considered to be less toxic and free of side effects than synthetic ones. Some work demonstrated that plants either contain antimicrobials that can operate in synergy with antibiotics or possess compounds that have no intrinsic antibacterial activity but are able to sensitize the pathogen to a previously ineffective antibiotic [3, 4]. Some plants can be sources of compounds that can potentiate the activity of antibiotics against resistant bacterial pathogens [5–9]. These compounds are believed to play a role in the plant's defense against infection by working in synergy with intrinsic antimicrobials. It has been suggested recently that such compounds can potentially be used to improve the efficacy of antibiotics against bacterial pathogens.

*Salvia* is an important genus widely cultivated and used in flavoring and folk medicines.* Salvia* species are used as traditional medicines all around the world, possessing antibacterial, antioxidant, anti-inflammatory, and analgesic properties [10].* S. sclarea*, popularly known as clary sage, is a biennial or perennial herb with diverse biological activities manifested by different components, mainly of EO.* Salvia* and other plants such as thyme, lavender, sage, basil, coleus, hyssop, and skullcap belong to the large plant family Lamiaceae [11]. Plants from Lamiaceae family are known for high content of EOs.

Biological properties of EOs and their antimicrobial activity have been attributed to their main compounds such as mono-, di-, and sesquiterpenes and a variety of low molecular weight aliphatic hydrocarbons, acids, alcohols, aldehydes, acyclic esters or lactones, coumarins, and homologues of phenylpropanoids [12]. These compounds have hydrophobic characteristics and interact with different sites of microbial cell, namely, with cytoplasmic membrane. They affect the activities of membrane associated enzymes; certain components of EOs can act as uncouplers, which interfere with proton translocation over a membrane vesicle and subsequently interrupt ADP phosphorylation. Specific terpenoids with functional groups, for example, phenolic alcohols or aldehydes, also interfere with membrane integrated or associated proteins, stopping their production, or activity EOs are also able to inhibit the synthesis of DNA, RNA, proteins, and polysaccharides in fungal and bacterial cells [13, 14]. The effect of EO may be associated with the cell envelope by interfering with the rigidity and integrity of the membrane, thereby changing the expression or function of cell wall-related genes such as penicillin-binding proteins (PBPs) [15].

Thymol, a* p*-cymene derived compound primarily found in some EO, suppresses the toxic shock syndrome toxin (TSST-1) secretion in* S. aureus* [16] and decreases the production of *α*-hemolysin, SEA, and SEB enterotoxins in* S. aureus* [17]. Similar effects were described for perilla oil [18]. Diverse spectrum of EOs (*S. officinalis*,* R. officinalis*,* A. alba*, and* E. caryophyllata*) as well as some of their major compounds (limonene, eugenol, and eucalyptol) inhibited QS gene expression in* S. aureus* [19]. Extract from* Rhus javanica* showed the inhibition of the genetic expression of virulence factors such as* sea*,* agr*A, and* sar*A in methicillin resistant* S. aureus* [20], as well as* mec*A gene, which is responsible for staphylococcal resistance to *β*-lactam antibiotics.

*Staphylococcus epidermidis* was previously regarded as an innocuous commensal microorganism on the human skin and mucous membranes [21]. However, nowadays it is seen as an important opportunistic pathogen, one of the most prevalent causes of nosocomial infections associated with newborn, severely ill, and immunocompromised patients, and is also frequently isolated from postsurgical infections, especially in association with indwelling prosthetic devices [22]. It was found that approximately 70% of the* S. epidermidis* strains circulating in the hospital environment are resistant to methicillin and that the majority of them are also resistant to other antimicrobial classes [23]. Resistance to methicillin is at 75–90% among hospital isolates of* S. epidermidis*, which is even higher than the corresponding rate for* S. aureus* (40–60%) [24].

Resistance to *β*-lactam antibiotics in* S. epidermidis*, similarly as in* S. aureus*, is mediated by (i) production of *β*-lactamase which hydrolytically destroys the *β*-lactam antibiotics and (ii) the acquisition of* mec*A gene that produces alternative PBP2, which have low affinity to *β*-lactam antibiotics. Gene* mec*A is carried on a chromosomal genetic element designated as staphylococcal chromosomal cassette* mec* (SCC*mec*). Currently, there are several different types of SCC*mec* elements, with several subtypes, characterized by a unique combination of the* mec* and the recombinase-encoding* ccr* gene complexes [25]. Some reports suggest that, in coagulase negative staphylococci, namely, in* S. epidermidis*, SCC*mec* structures are more diverse and include either* mec-ccr* combinations not yet described for* S. aureus* [23] or more than one* ccr* allotype [26]. In* S. epidermidis* prevail SCC*mec* type IVa [27] and SCC*mec* type III [28]. Miragaia et al. [23] characterized 139 isolates of MRSE and found that 41% of the isolates harbored SCC*mec* type IV and 27% carried SCC*mec* type III, while SCC*mec* types V, I, and II were presented only in 6%, 4%, and 4% of the isolates, respectively. Of the 44 MRSE isolates recovered from the blood of patients with prosthetic valve endocarditis, 2% harbored SCC*mec* type I, 34% harbored type II, 28% harbored type III, and 36% harbored type IV [29].

Expression of* mec*A is inducible and can be controlled by either its cognate regulators MecI (DNA binding repressor protein) and MecR1 (sensor/signal transducer) or the structurally and functionally similar *β*-lactamase regulators BlaI and BlaR1, respectively [30]. The transcription of* mec*A and* bla*Z is corepressed by the regulators of the two regulons, MecI and BlaI. These regulators are almost identical and can replace each other [31] and both MecI and BlaI can bind as homodimers to the promoter/operator region of both* mec*A and* bla*Z [32]. In the presence of antibiotics, the sensing is mediated by signal transduction via the two transmembrane inducers, MecR1 or BlaR1, which will result in proteolytic autocleavage of the cytoplasmic domains of these proteins [33]. To perform this autocleavage, the transducers undergo acylation by the antibiotic that causes conformational changes in the molecule [34]. Autocleavage of the signal transducer is followed by cleavage of the cognate repressor, MecI or BlaI, and by subsequent induction of the transcription of* mec*A or* bla*Z [33].

In our previous work [35] we showed synergistic effects of oxacillin with plant extracts and EOs from several plant species of Lamiaceae family. The purpose of the present study was to determine the effect of EO from* S. sclarea* on the expression of* mec*A gene in strains* S. epidermidis* possessing different types of SCC*mec* and determine synergistic effect of oxacillin and EO in these strains.

## 2. Material and Methods

### 2.1. Plant Material and EO Isolation

The aerial parts of* S. sclarea* were harvested at the optimal growing and development stage. The EO was prepared in accordance with the European Pharmacopoeia [36]. The plant material was air-dried and submitted to hydrodistillation for 4 h. Isolated oil was diluted in n-hexane and dried over anhydrous sodium sulfate. The most frequent EO components were determined by the GC method.

### 2.2. Bacterial Strains

MRSE strains were obtained from clinical samples of patients with positive hemocultures from the University Teaching Hospital Old Town, Bratislava, Slovak Republic, and were kindly provided by Dr. Slobodníková from the Institute of Microbiology, Faculty of Medicine, Comenius University, in Bratislava, Slovak Republic.

### 2.3. Determination of Oxacillin Resistance and Genes from* mec* and* bla* Operons

The isolates were screened for their susceptibility towards oxacillin using disc diffusion method on Mueller-Hinton agar (HiMedia, India) in accordance with the Clinical and Laboratory Standard Institute Guidelines (CLSI) standards [37]. Suspension of the tested bacteria (0.1 mL of 10^8^ cells/mL) was spread onto solid media plates. Antimicrobial susceptibility test discs (HiMedia, India) with ampicillin (10 *μ*g) and oxacillin (1 *μ*g) were placed on the incubated plates. These plates, after 2 h of maintenance at 4°C, were incubated for 24 h at 37°C and the diameters of the resulting zones of inhibition were measured in millimeters.

Extraction of genomic DNA was performed by using DNeasy Blood and Tissue Kit (Qiagen, Germany) according to the manufacturer's instructions. All the isolates were tested for genes of the* mec* and* bla* operon using the polymerase chain reaction method. Each PCR sample contained 2.5 *μ*L of 10x Star Taq enzyme buffer (Gene Craft, Germany), 0.2 mM of each deoxynucleoside triphosphate (Gene Craft, Germany), 0.2 *μ*M of each of the forward and reverse primers, 1.5 U* Taq* DNA polymerase (Gene Craft, Germany), and 2 *μ*L template DNA. The PCR primers were designed by Primer3 and Primer-Blast and synthesized by Microsynth (Switzerland). Details of primer sequences are listed in Table 1. The cycling conditions were as follows: preheating for 5 minutes at 94°C, followed by 30 cycles of denaturation at 94°C/30 seconds, annealing at 55°C/40 seconds, extension at 72°C/30 seconds, and final extension for 5 minutes at 72°C. PCR amplicons were analyzed by 2% agarose gel electrophoresis and stained with ethidium bromide.

### 2.4. SCC*mec* Typization

The SCC*mec* type was determined using the protocol and primers proposed by Zhang et al. [38]. Multiplex PCR was performed in 25 *μ*L reactions with 2.5 *μ*L of 10x Star Taq enzyme buffer (Gene Craft, Germany), 0.2 mM of each deoxynucleoside triphosphate (Gene Craft, Germany), various concentrations of the respective primers [38], 1.0 U of* Taq* DNA polymerase (Gene Craft, Germany), and 2 *μ*L of template DNA. The amplification was performed using a Biometra Thermal Cycler (Germany) with an initial denaturation step at 94°C/5 min followed by 30 cycles of denaturation at 94°C/1 min, annealing at 55°C/1 min, extension at 72°C/2 min, and a final extension step at 72°C/5 min. PCR products were detected on a 2% agarose gel and stained with ethidium bromide.

### 2.5. Determination of Minimal Inhibitory Concentration (MIC)

Minimal inhibitory concentrations (MIC) for oxacillin (Sigma-Aldrich) and EO (prepared as stated above) were determined by standard broth microdilution method using 96-well microtiter plates in accordance with CLSI [37] in Mueller-Hinton broth. The* S. epidermidis* suspension was adjusted to the 0.5 McFarland standard and diluted to obtain a final turbidity in wells approximately 1 × 10^6^ CFU/mL. 0.1 mL of bacterial suspension was mixed with an equal volume of each dilution of oxacillin or EO, and optical density (OD_600_) values were determined after 24 hours of growth at 37°C using a 96-well plate reader Varioskan Flash (Thermo Fisher Scientific, Finland). The MIC was defined as the lowest concentration of antimicrobial agent that completely inhibits growth of the organism.

### 2.6. The Checkerboard Method

The study of the interaction between EO and oxacillin was done using the checkerboard method. Twofold serial dilutions of oxacillin prepared in horizontal rows of 96-well microtiter plate were subsequently cross-diluted vertically by twofold serial dilutions of EO. Microtiter plates were inoculated with test organism and incubated for 24 h at 37°C. MIC values of the combinations were determined as the lowest concentration that completely inhibited bacterial growth recorded as the optical density at 600 nm using Varioskan Flash (Thermo Fisher Scientific, Finland). Interaction between EO and oxacillin was then determined by calculating the fractional inhibitory concentration (FIC) indices. The FICI is defined as follows: MIC of substance A tested in combination/MIC of substance A tested alone + MIC of substance B tested in combination/MIC of substance B tested alone. The FICI is interpreted as follows: FICI < 0.5, synergistic effect; 0.5 < FIC < 1, additive effect; 1 < FICI < 4, indifferent effect; and FIC > 4, antagonistic effect [39]. The synergistic effect is shown graphically by applying isobole method. The shape of the isobologram curve can be convex, linear, or concave, which is indicative of the synergistic, indifferent, and antagonistic interactions, respectively [40].

### 2.7. The Time-Kill Assay

The time-kill assay was carried out in order to determine antibacterial and potential synergistic effects of EO when used singly and in combination with oxacillin. Bacteria (5 × 10^5^ CFU/mL) were exposed to oxacillin and EO alone or in combination in concentrations 1/2 MIC and incubated at 37°C. Aliquots (0.1 mL) were taken at 0, 6, 10, and 24 h and diluted in normal saline as needed to enumerate 30–300 colonies. The diluted cultures were spread thoroughly on plates containing Mueller-Hinton agar. After incubating at 37°C for 24 h, the growing colonies were counted. The experiment was performed in triplicate; data are shown as mean ± standard deviation from three independent experiments.

### 2.8. RNA Isolation and Real-Time PCR

MRSE strains were treated with various concentrations of oxacillin and EO alone or with their combination for 30 minutes. Total RNA was isolated by using RNeasy Mini Kit (Qiagen, Germany) according to the manufacturer's instructions. DNA contamination from the total RNA preparations was removed with on-column RNase-Free DNase treatment (Qiagen, Germany). Measuring A_260_/A_280 _nm ratio assessed the nucleic acid purity. To generate cDNA, total RNA was reverse transcribed using the GoTaq 2-Step RT-qPCR System (Promega) with specific primers (Table 1). Real-time PCR was performed at least three times for each examined gene with an Applied Biosystem 7500 FAST Real-Time PCR System. The Ct values and the qPCR were normalized to the housekeeping gene for glyceraldehyde-3-phosphate dehydrogenase (GAPDH) using the 2^−ΔΔCt^ method [41].

### 2.9. Statistical Analysis

The experimental data are expressed as the mean ± standard deviation (±SD) from three independent experiments. Statistical analyses were performed using the *t*-test. *P* < 0.05 was considered to indicate a statistically significant difference.

## 3. Results and Discussion

### 3.1. Essential Oil

The essential oil obtained from aerial parts of* S. sclarea* was analyzed by gas chromatography (GC) and 12 compounds representing 75.2% of the essential oil were identified. The main components were linalyl acetate (38.67%), linalool (20.42%), germacrene (5.31%), geraniol (1.42%), and *β*-caryophyllene (1.80%).

### 3.2. Isolate Selection for Further Analysis

In our previous work we showed that addition of plant extracts and EOs from some species of* Salvia* plants increases the susceptibility of MRSE to oxacillin. EOs and oxacillin in combination showed synergistic effect, inhibited growth, and even killed the bacteria [35].

In this study we used 30 clinical isolates of MRSE for study of potential synergistic effects of EO from* S. sclarea* and oxacillin, and by using checkerboard method we confirmed this effect in 23 isolates (76.7%) (FICI from 0.125 to 0.381). The remaining 7 strains (23.3%) showed additive effect (FICI from 0.531 to 0.793). None of the strains showed antagonistic effect.

**Figure 1:**
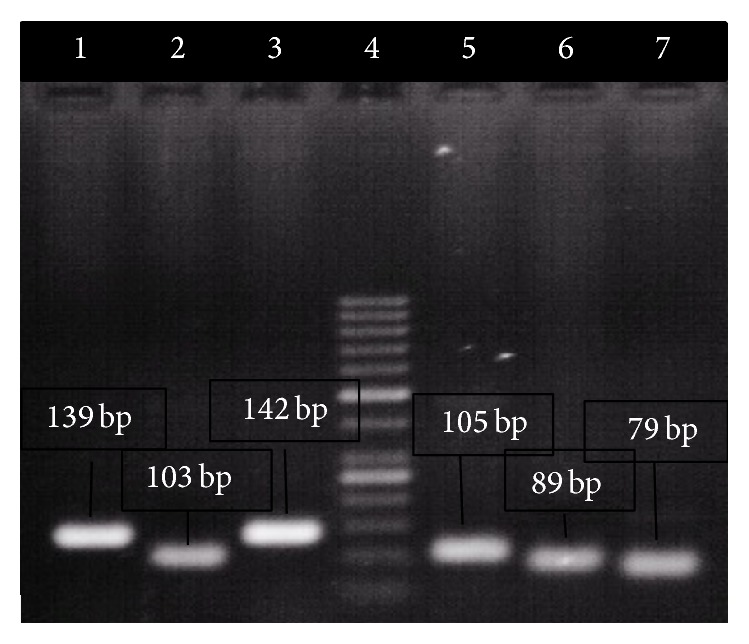
Detection of genes from* mec* and* bla* operons in strain R8. Lane 1:* mec*A, lane 2:* mec*I, lane 3:* mec*R1, lane 4: 100 bp marker, lane 5:* bla*Z, lane 6:* bla*I, and lane 7:* bla*R1.

**Figure 2:**
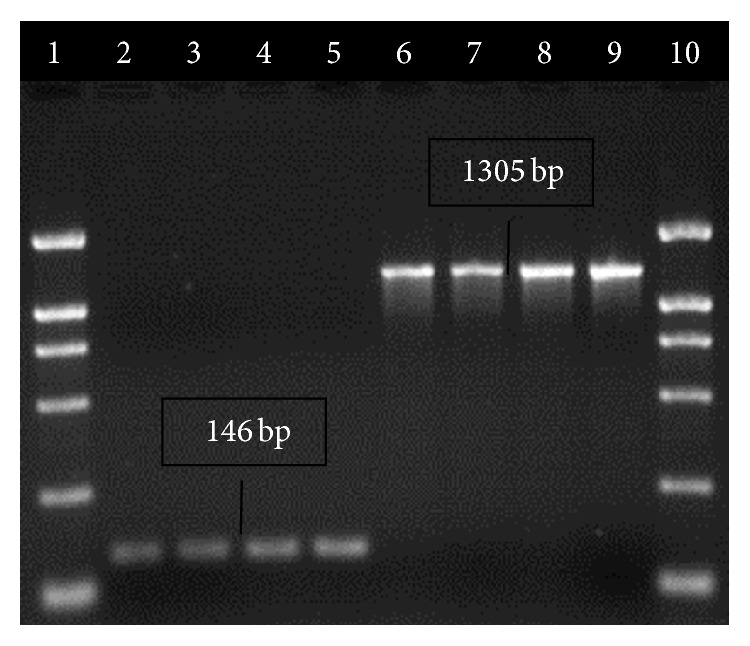
Detection of* mec* class. Lane 1: 2000 bp marker, lanes 2, 3: strain R8, lanes 4, 5: strain R22 (class A), lanes 6, 7: strain R17, lanes 8, 9: strain R12 (class B), and lane 10: 2000 bp marker.

**Figure 3:**
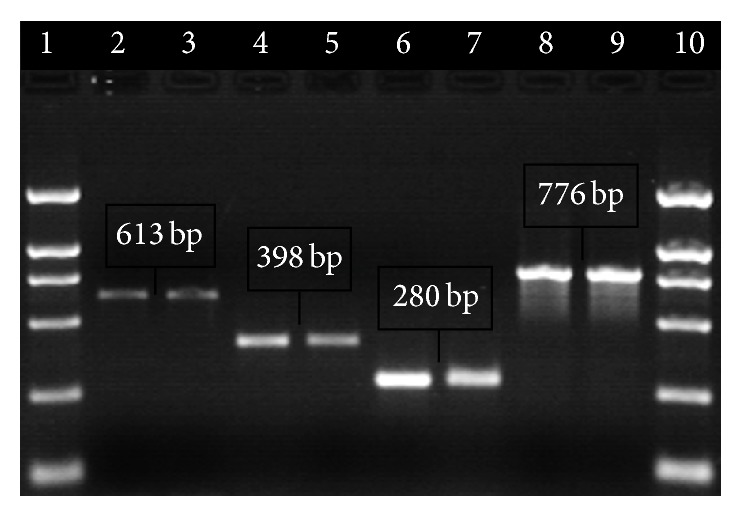
Typization of SCC*mec* in selected strains. Lane 1: 2000 bp marker, lanes 2, 3: SCC*mec* type I (strain R17), lanes 4, 5: SCC*mec* type II (strain R8), lanes 6, 7: SCC*mec* type III (strain R22), lanes 8, 9: SCC*mec* type IVa (strain R12), and lane 10: 2000 bp marker.

Based upon the phenotype analysis of methicillin resistance (MIC of oxacillin, Table 2), screening for presence or absence of* mec*A,* mec*I,* mec*R1,* bla*Z,* bla*I, and* bla*R1 gene using PCR (Figure 1), and analysis of* mec* class (Figure 2) and on SCC*mec* typization we selected four strains possessing different SCC*mec* types I, II, III, and IVa (Figure 3), which cover all SCC*mec* types and all variations of* mec* and* bla* operons present in collection of isolates.

### 3.3. Real-Time PCR Analysis of Expression of* mec*A Gene

We have attempted to assess if EO alone or in combination with oxacillin influences the expression of* mec*A gene. Firstly, we characterize the* mec*A/*mec*I/*mec*R1 and* bla*Z/*bla*I/*bla*R1 regions of our strains and determine the effect of presence or absence of* mec* and* bla* elements on the MIC of oxacillin. The characteristics of these strains are in Table 2. Isolates R17 and R12 which possess SCC*mec* types I and IVa, respectively, and class B* mec* have genotypes +*bla*I/+*bla*R1 and −*mec*I/*mutmec*R1 (absent* mec*I and truncation of* mec*R1 by insertion of IS*1272*). These strains showed high resistance, correlating with the fact that* mec*I deletion can lead to increased resistance [42]. Isolate R8 (SCC*mec* type II) has both complete repressor/sensor systems +*mec*I/+*mec*R1 and +*bla*I/+*bla*R1. Isolate R22 (SCC*mec* type III) has +*mec*I/+*mec*R1 system but this isolate was the only one in which we were unable to confirm the presence of the* bla* operon. This isolate has the lowest MIC of oxacillin from four isolates. Hackbarth et al. described that interruption of* bla*R1 results in constitutive repression and therefore decreased resistance [43].

**Figure 4:**
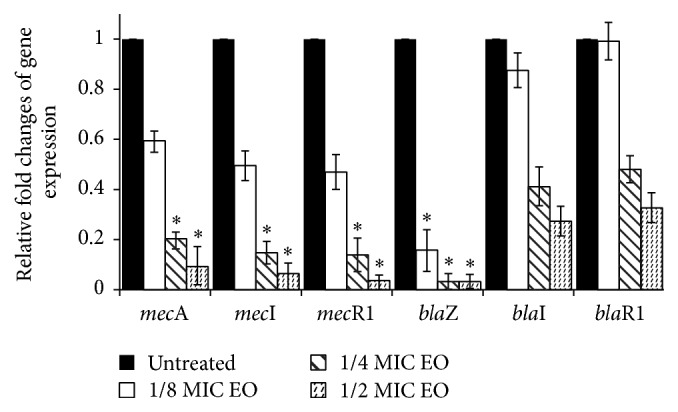
Relative expression of genes of* mec* and* bla* operons.* S. epidermidis* R8 was treated with subinhibitory concentrations of EO from* S. sclarea* for 30 min. Transcript levels were monitored by real-time PCR as described in the text. Using the 2^−ΔΔCt^ method, the data are presented as the fold change in gene expression normalized to an endogenous reference gene (GAPDH) and relative to the untreated control (value 1). Values represent the mean ± SD for three independent experiments. ^*∗*^ *P* < 0.05.

We used real-time PCR to investigate the expression of genes from* mec* and* bla* operons and to determine the influence of EO on their expression. Quantification data for all genes were normalized to the reference gene for glyceraldehyde-3-phosphate dehydrogenase (GAPDH). In Figure 4 is relative expression of genes in strain R8, which have complete both operons. Regardless of constitutive or inducible expression of genes our data show that 30 min treatment of culture with subinhibitory concentrations of EO from* S. sclarea* leads to decreasing of expression of all genes from both operons. It is known that EOs influence membranes and proteins in cytoplasmic membrane [44]; therefore we can assume that they can influence conformation of MecR1, or BlaR1, which can have impact on transcription of both operons. While EO showed similar and dose dependent effect on single genes from* mec* operon, in case of* bla* genes the effect of EO on* bla*I and* bla*R1 gene expression was weaker. However the expression of gene* bla*Z was decreased the most from all genes. Similar results, the inhibition of expression of resistant genes* mec*A,* mec*I, and* mec*R1, against methicillin resistant* S. aureus* by hexane and chloroform fractions of* Salvia miltiorrhiza* Bunge, were described by Lee et al. [45].

**Figure 5:**
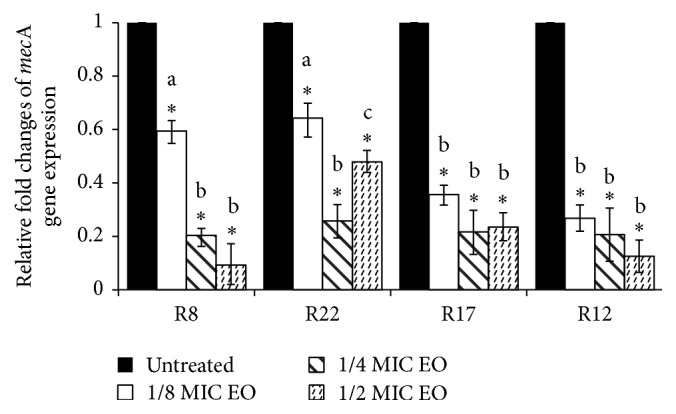
Relative expression of* mec*A gene in* S. epidermidis* strains with different types of SCC*mec*. Strains were treated with subinhibitory concentrations of EO from* S. sclarea* for 30 min. Using the 2^−ΔΔCt^ method, the data are presented as the fold change in gene expression normalized to an endogenous reference gene (GAPDH) and relative to the untreated control (value 1). Values represent the mean ± SD for three independent experiments. ^*∗*^ *P* < 0.05. Different letters signify statistical differences between values (*P* < 0.05).

One isolate from each SCC*mec* type was exposed to increasing amount of EO and as is shown in Figure 5, EO reduced the expression of* mec*A gene in all strains. Regarding SCC*mec* type and genetic background of* mec* and* bla* operons, strains R8 and R22 have* mec* A class with all three* mec* genes, while strains R17 and R12 have* mec* B class (absent* mec*I and truncation of* mec*R1 by insertion of IS*1272*). As is obvious from Figure 5, we did not find significant differences in expression of* mec*A gene between strains with different* mec* class after treatment with EO at concentrations 1/4 and 1/2 of MIC; however in concentration 1/8 MIC there was statistically significant (*P* < 0.05) difference between strains of both classes of* mec* complex, when EO decreased the expression of* mec*A gene more in strains R17 and R12 in the comparison with strains R8 and R22. We estimated the expression of* mec*A gene in the presence of EO also in three random selected strains with IVa type of SCC*mec* and the results were similar (data not shown).

**Figure 6:**
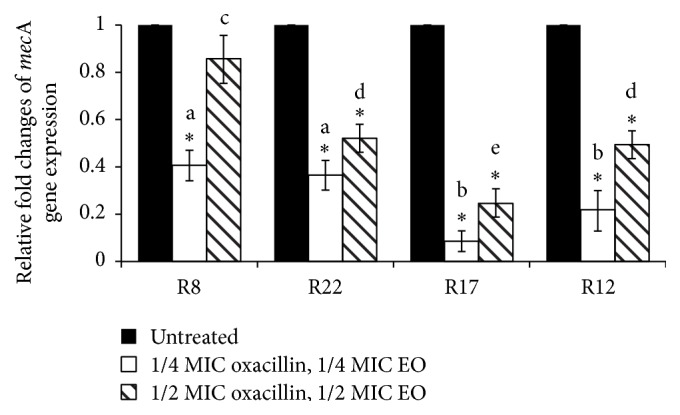
Relative expression of* mec*A gene in* S. epidermidis* strains with different types of SCC*mec* after treatment with EO from* S. sclarea* and oxacillin. Using the 2^−ΔΔCt^ method, the data are presented as the fold change in gene expression normalized to an endogenous reference gene (GAPDH) and relative to the untreated control (value 1). Values represent the mean ± SD for three independent experiments. ^*∗*^ *P* < 0.05. Different letters signify statistical differences between values (*P* < 0.05).

Finally we examined the expression of* mec*A gene after 30 min treatment with oxacillin and EO. Oxacillin alone induced higher expression of* mec*A gene (data not shown). The addition of EO not only inhibited the induction of expression, but also reduced the expression of* mec*A gene in the comparison with untreated control (Figure 6). The combination of 1/4 MIC of both compounds was more effective, probably due to lower concentration of oxacillin and lower induction of expression of* mec*A gene. We found similar trend as in the case of EO alone, that is, higher reduction of expression in strains R17 and R12 in the comparison with strains R8 and R22.

### 3.4. Modulatory Study

**Figure 7:**
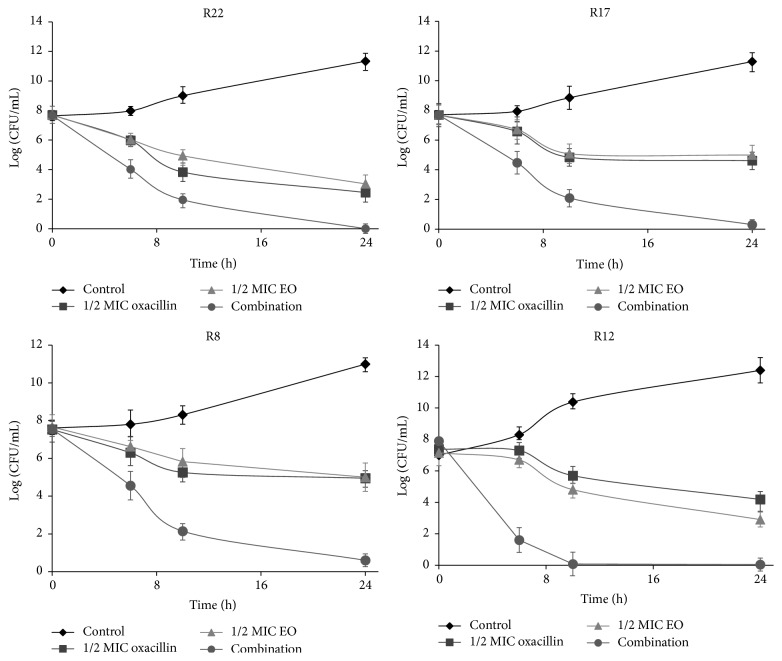
Time-kill curves of strains after treatment with 1/2 MIC of oxacillin and 1/2 MIC of EO alone or in combination.

The viable cell counts for four isolates of MRSE differing in type of SCC*mec* after exposure to 1/2 MIC of oxacillin and EO alone and in combination at different times are shown in Figure 7. The 1/2 MIC values were specific for each particular strain. The combination of 1/2 MIC EO + 1/2 MIC oxacillin completely inhibited the growth of all four strains after 24 h. The same combination caused an over 3 log_10_-fold reduction in the bacterial count yet after 10 h (strains R17 and R8) in comparison with the most active compound alone. In strain R12 the reduction was more than 5 log_10_-fold in 6 h and from 10 h the growth of this strain was completely inhibited.

**Figure 8:**
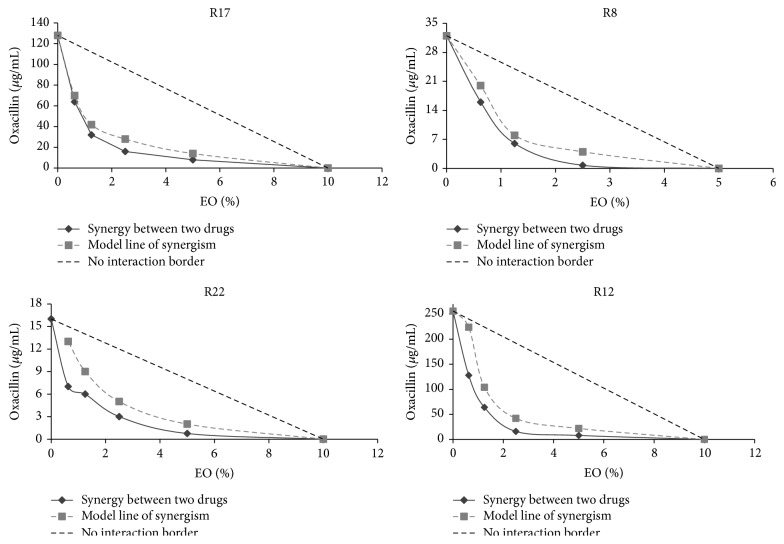
Isobole curves revealing the synergistic effect of* S. sclarea* EO with oxacillin against* S. epidermidis* strains with different types of SCC*mec.*

To get a better understanding of the modulatory effect of EO we examined the interaction between EO and oxacillin via the checkerboard method and described it in terms of fractional inhibitory concentration (FIC) indices. The FIC indices of EO in combination with oxacillin were 0.381 for strain R17, 0.156 for R8, 0.125 for R22, and 0.376 for R12, which according to Pillai et al. [39] indicate synergistic interactions. Combinatorial profiles are presented graphically in Figure 8. The synergistic interaction can be read according to the curve indicating borderline synergy according to FIC 0.5. Isobolograms of all strains have convex shape beyond the borderline of synergisms and clearly show the potentiating effect of* S. sclarea* EO on oxacillin susceptibility in these strains.

## 4. Conclusion

Our data reveal the potential of EO from* S. sclarea* to be candidate for combination therapy against MRSE, because it has synergistic effect with oxacillin in all tested strains. The killing effect of the combinatorial treatment is connected with reduction of expression of* mec*A gene and other genes participating in staphylococcal resistance to *β*-lactams antibiotics. Lower expression of these genes reduces the two major bacterial defense mechanisms against *β*-lactam antibiotics. Although synergistic effect of EO may be caused by several mechanisms, observed different levels of the reduction of* mec*A expression in strains with different types SCC*mec* and different genetic background in* mec* and* bla* operons confirmed that reduction of* mec*A gene expression can be one of major mechanisms.

## Figures and Tables

**Table 1:** Primer sequences (5′ to 3′) used for PCR and real-time PCR.

Primers	Sequences (5′-3′)	Product length (bp)
GAPDH		
Forward	TCAACGATTTAACAGATGACGCA	77
Reverse	TTCGTCTTTGAAACGACCTTGTG	
*mecA*		
Forward	TCCACCCTCAAACAGGTGAA	139
Reverse	TGGAACTTGTTGAGCAGAGGT	
*mecI*		
Forward	TCATCTGCAGAATGGGAAGTT	103
Reverse	TTGGACTCCAGTCCTTTTGC	
*mecR1*		
Forward	AGCACCGTTACTATCTGCACA	142
Reverse	AGAATAAGCTTGCTCCCGTTCA	
*blaZ*		
Forward	TCCTAAGGGCCAATCTGAACC	105
Reverse	ACACTCTTGGCGGTTTCACT	
*blaI*		
Forward	ACTGTATGGAGGGGACATGAA	89
Reverse	TGTCTCGCAATTCTTCAATTTCTT	
*blaR1*		
Forward	GCCCTTACACAACGATTACCAA	79
Reverse	GCTGTACATGACGAAAGATCCAC	

**Table 2:** Profile of *mec* and *bla* elements in *S*. *epidermidis* strains used in the work.

Strain	*mec*A	*mec*I	*mec*R1	*bla*Z	*bla*I	*blaR*1	SCC*mec* type	Mec class	MIC oxa. (*μ*g/mL)
R17	+	−	−	+	+	+	I	B	128
R8	+	+	+	+	+	+	II	A	32
R22	+	+	+	−	−	−	III	A	16
R12	+	−	−	+	+	+	IVa	B	256
